# Characteristics of ABO Antibodies in Group O Malaysian Blood Donors

**DOI:** 10.21315/mjms2023.30.4.6

**Published:** 2023-08-24

**Authors:** Nur Ilyia Syazwani Saidin, Noor Haslina Mohd Noor, Shafini Mohamed Yusoff, Mohd Shafiq Sauli

**Affiliations:** 1 Department of Haematology and Transfusion Medicine Unit, School of Medical Sciences, Universiti Sains Malaysia Kelantan, Malaysia; 2 Hospital Universiti Sains Malaysia, Kelantan, Malaysia

**Keywords:** anti-A, anti-B, antibody titres, haemolytic transfusion reaction, O group, haemolysin

## Abstract

**Background:**

Haemolytic transfusion reactions (HTRs) due to anti-A and anti-B antibodies in Group O blood products are rare but potentially fatal. This study aimed to identify the prevalence of high ABO antibody titre and the immunoglobulin (Ig) classes (IgM only or with IgG) and the prevalence of haemolysin antibodies in Group O blood donors.

**Methods:**

Plasma from Group O blood donors was tested by using antibody titration at room temperature. Titres ≥ 64 were considered high. The plasma was treated with 0.01 M dithiothreitol (DTT) to determine the presence of IgG antibodies and titre. IgG titres ≥ 64 were considered high. Tests for haemolysis were conducted by mixing the plasma with 3% fresh A1 and B cell suspensions and incubating at 37 °C. The haemolysis was observed macroscopically.

**Results:**

Of 311 donors, 238 (76.5%) showed high anti-A and/or anti-B antibody titres. The highest antibody titre obtained was 256. Female and younger donors (< 40 years old) had higher anti-A and anti-B titres. The anti-B titre showed an association with gender (*P* < 0.001), and was high in female donors (77.8%). Males aged over 50 years old were found to have low mean titre antibodies. Most donors had both IgM and IgG ABO antibodies. The prevalence of haemolysins in our population was 3.5%.

**Conclusion:**

Most of our O blood donors had a high ABO antibody titre but a low prevalence of haemolysins. Males aged over 50 years old are the best O donors for preventing HTRs, particularly when mismatch transfusion is required. We recommend a transfusion unit screen for ABO antibody titre in younger female donors (< 40 years old), to prevent the transfusion of high titre O blood products into non-O recipients.

## Introduction

In Malaysia, the Malays and Chinese have the highest percentage of blood Group O (36.7%). Among Indians, O and B blood groups were found at equivalent frequencies (1). Blood Group O is well known as the universal blood group because its red cells lack A and B antigens (2). Thus, it is widely accepted as safe to transfuse Group O red cells to non-O group recipients, particularly in life-saving conditions (3). However, its plasma contains anti-A and anti-B antibodies of the immunoglobulin M (IgM) type. A small proportion of these antibodies can be IgG or IgA. Both ABO IgM and IgG antibodies can activate complement and result in intravascular haemolysis (4). These may occur following the transfer of ABO antibodies into non-O recipients, such as in ABO-incompatible platelet transfusions (5).

ABO antibodies begin to be produced at birth, although the titre is typically too low for detection. The titre levels are variable, depending on gender, age and environmental factors. Among blood Group O donors, anti-A titres are frequently reported to be greater than anti-B titres (6). The ABO IgM antibody titre ≥ 64 has been agreed upon in many works of literature as high, whereas the high titre for IgG antibodies is ≥ 200 (7–9).

During the Vietnam War, the United States military force used whole-blood products with a titre of < 256 to treat haemorrhagic shock (10). In the United Kingdom, antibody titration is required for all O group donors, with ABO antibody titres > 100 deemed as high and allowing transfusion to only O blood group patients (7).

Standard guidelines for defining the high titre of ABO antibodies are lacking in the transfusion community. For both ABO IgM and IgG antibodies, our study used a cut-off titre value of ≥ 64 as a high titre. This cut-off point was selected considering other studies that employed a comparable cut-off titre in their research (7, 11).

This study aimed to estimate the prevalence of the high titre of ABO antibodies among O blood donors in our local population and its association with gender and age. We also aimed to determine the type of Igs of anti-A and anti-B antibodies, either IgM only or a combination of IgM and IgG types. Moreover, we intended to identify the prevalence of a high IgG titre and haemolysin antibodies among our Group O blood donors. The Group O blood product is widely used in clinical transfusion practice. Thus, we hope that the findings of this study contribute to safe transfusion practices. To the best of our knowledge, no similar research has been performed on our local population.

## Methods

### Study Setting

The study was conducted at the Transfusion Medicine Unit of Hospital Universiti Sains Malaysia from January to December 2020. The study protocol was approved by the Institutional Review Board of the Human Research Ethics Committee.

### Study Population

A total of 311 samples were collected randomly from whole-blood Group O donors, from 18 years old to 60 years old of age. All selected donors fulfilled the pre-donation criteria according to the national guidelines for donor selections.

### Samples and Reagents

About 3 mL of blood was collected into an ethylene-diamine-tetra acetic acid tube. The sample was stored in a 4 °C–6 °C fridge and analysed less than 48 h after collection. In-house preparations of 3% concentrated A1 cells and B cells were used for titre studies, and Ig and haemolysis tests. To cleave pentameric IgM disulphide bonds, 0.01 M dithiothreitol (DTT) (Sigma-Aldrich D9163) was used. DTT eliminated the agglutinating reactivity of IgM and permitted the detection of IgG antibodies in the plasma (12).

### Titration Procedure

Titration procedures were performed to assess anti-A and anti-B titres according to the method described by the Technical Manual of the American Association of Blood Banks (AABB) (13). The plasma sample was mixed with 0.9% saline in the glass tube and two sets of serials with twofold doubling dilutions were prepared. One drop of A1 cell and B cell suspension was added to the diluted plasma, respectively. The test mixture was incubated at room temperature for 10 min and centrifuged at 3,400 rpm for 15 sec. The highest dilution causing agglutination (macroscopic observation = 1+ reaction) was presumed to represent the IgM antibody titre (14). The antibody titre of ≥ 64 was considered high.

### Determination of Immunoglobulin Classes

The presence of IgG was detected by comparing the reactivity of treated plasma with DTT and untreated plasma in phosphate-buffered saline (PBS) using a glass tube. Serial dilutions were prepared from treated plasma with DTT and incubated for 60 min at 37 °C. Serial dilutions of untreated plasma in PBS were incubated for 30 min at 37 °C. Four drops of each dilution of treated and untreated plasma were mixed with one drop of A1 cells and B cells in a new set of tubes and then centrifuged. The agglutination observed for each dilution was graded. Equivalent reactivity in both treated and untreated plasma indicates the presence of IgG antibodies. The IgG titres were represented by the highest dilution with observed agglutination, and a titre of ≥ 64 was considered high. The lack of reactivity in the treated plasma indicates IgM antibodies only. The procedure and interpretation were adapted from the Technical Manual of the AABB (15).

### Haemolysin Tests

For the haemolysin tests, 100 μL of test plasma was dispensed into tubes labelled A and B. Another 100 μL of A1 cells and B cells were added to the tubes labelled A and B, respectively. The mixture was then incubated at 37 °C for 60 min, followed by centrifugation. The presence of haemolysis was observed macroscopically according to the Clinical Laboratory Standard Institute guidelines. Negative haemolysis was confirmed by spectrophotometry measurements, defined by free haemoglobin in plasma < 0.5 g/L (16).

### Statistical Analyses

Descriptive statistics were used to describe gender, age, ethnicity, antibody titre, Ig classes and haemolysin. Statistical analyses were performed by using the Pearson chi-squared test or Fisher’s exact test to determine the association of antibody titre and haemolysis, with gender and age. A *P*-value < 0.05 was considered statistically significant. All datasets were analysed by using standard statistical software (SPSS version 25.0).

## Results

Demographic data from the 311 selected Group O blood donors showed that 185 (59.5%) were males and 126 (40.5%) were females. The largest ethnic group was Malay, 273 (87.8%), followed by Chinese, 33 (10.6%); Siamese, 4 (1.3%) and Indian, 1 (0.3%). Among age groups, those aged 18 years old–29 years old accounted for the highest number of donors, with 144 (46.3%); followed by 30 years old–39 years old, with 92 (29.6%); 40 years old–49 years old, with 43 (13.8%); and ≥ 50 years old, with 32 (10.3%). The mean age of our study population was 32.5 years old.

### Anti-A and Anti-B Titres

Of the 311 donors, 238 (76.5%) donors had high anti-A and/or anti-B titres (≥ 64). There were 32 (13.5%) donors with high anti-A titre, 26 (10.9%) donors with high anti-B titre, and 180 (75.6%) donors with both high anti-A and anti-B titres. There were slightly more donors with higher anti-A titres than anti-B titres. The mean of anti-A and anti-B titres were 68 and 67, respectively. The highest antibody titre obtained was 256 for both anti-A and anti-B antibodies.

We found that most female donors showed high anti-A and anti-B titres. Of the 126 female donors, 90 (71.4%) showed high anti-A titres and 98 (77.7%) showed high anti-B titres. These donors may have had a high titre for anti-A or anti-B alone or a high titre for both antibodies. Among the female donors, a high antibody titre was found more in younger age groups (< 40 years old) (Table 1). Of the 185 male donors, 122 (65.9%) exhibited high anti-A titres and 108 (58.3%) exhibited high anti-B titres. Male donors aged less than 40 years tended to have high antibody titres, similar to female donors (Table 1).

We looked at how gender and age affected the ABO antibody titre. The anti-A titre and gender did not correlate statistically. The anti-B titre, however, demonstrated a relationship with gender (*P* = 0.001). A high anti-B titre was present in the majority of female donors (77.2%). Age and antibody titre did not correlate, according to our findings. We observed that donors aged > 50 years old have a lower chance of obtaining high titre antibodies than other age groups (Table 2).

### Immunoglobulin Classes

We discovered that a majority of donors (a total of 209) exhibited ABO antibodies of both the IgM and IgG subtypes. The presence of anti-A IgG was detected in 46 (22.0%) donors, whereas anti-B IgG was detected in 45 (21.5%) donors. IgG for both anti-A and anti-B antibodies were present in 118 donors (56.5%). Only IgM-type ABO antibodies were present in the 102 remaining donors.

### Anti-A and Anti-B Titres (IgG Types)

We discovered that 124 (59.3%) of the 209 donors with IgG-type antibodies had a high IgG titre (≥ 64) (Table 3). The IgG titre was between 32 and 256. Younger donors—those aged less than 40 years old—were more likely to have high IgG titres. We discovered a significant relationship (*P* = 0.002) between anti-B IgG titres and gender, with female donors having a higher likelihood of obtaining high anti-B IgG titres (Table 4). Anti-A IgG titres and gender were not significantly correlated, and anti-A IgG and anti-B IgG titres were not significantly correlated with age.

### Haemolysin Antibodies

Eleven (3.5%) donors were found to have haemolysin antibodies. Of these, four (1.3%) donors had haemolysin A, five (1.6%) donors had haemolysin B, and two (0.6%) donors had both haemolysins A and B. The details of the 11 donors with haemolysins are shown in Table 5. Haemolysin-positive donors tended to be male and aged younger than 40 years old. The majority of anti-B haemolysins were detected in male donors and were more common than anti-A haemolysins. Contrarily, anti-A haemolysins were more common in female donors. The majority of haemolysin-positive donors also showed high antibody titres (≥ 64) and IgG types. However, the prevalence of haemolysins did not correlate statistically with gender, age or antibody titres.

## Discussion

It is known that blood Group O containing high anti-A and anti-B titres could lead to haemolytic transfusion reactions (HTRs) after ABO-incompatible blood transfusion. A case of HTR after transfusion with O platelets containing a high titre of anti-A antibodies into a Group A recipient has been reported (17). These findings suggest that a mismatch transfusion of blood Group O is not entirely safe and haemolysis may be more common than appreciated.

In our study, we observed a high prevalence of donors with high anti-A and anti-B titres. According to a recent study conducted on an Indian population, the prevalence of donors with high anti-A and anti-B titres was 36.0% and 32.0%, respectively (18). In Thailand, Group O blood donors were more likely to have strong ABO antibodies, with anti-A and anti-B titres being high in 75.7% and 80.0% of donors, respectively (11). A study of 3,274 male Group O donors in South Texas discovered that 426 (13.0%) had high titre antibodies (19). In Boston, research on platelet apheresis Group O found that 25.0% of the components were classified as a high titre (20). All these findings imply that Group O donors may have high titre antibodies that could cause a transfusion reaction in the recipient.

In our study, the anti-A and anti-B titres ranged from 16 to 256. ABO antibody titres ranged from 16 to 128 in Brazilian O blood donors and from 4 to 128 in Korean blood donors (21, 22) anti-A and anti-B titres of Group O blood donors in Thailand ranged from 8 to 1,024 (11). Sood et al. (23) discovered even higher antibody titres, ranging from 2 to 4096, in Indian donors.

We explored the gender distribution of titres and noticed that female donors had higher mean antibody titres than male donors. Our study also found a high antibody titre in younger females, aged less than 40 years old. This is comparable to a study by Kannan et al. (18), who discovered a high titre of anti-A among female donors of age 30 years old–39 years old. These findings are possible as a result of immunisation and pregnancy during this reproductive age. Females can develop stronger humoral and adaptive immune responses than males, thus generating higher antibody levels (24). These are marked by higher basal and post-vaccination IgG levels and increased B-cell numbers in response to vaccination and viral infection (25). The chromosomal genetic differences, sex hormones and gender-specific differences in the microbiome are among the factors that influence the difference in humoral immunity between males and females. The X chromosome expresses 10 times more genes than the Y chromosome, and many genes on the X chromosome are known to influence immunity, thus affecting antibody production (26).

We analysed the age-wise distribution of antibody titres in O group donors. The mean donor age was 32.5 years old, and 46.3% of blood donors were aged between 18 years old and 29 years old. The highest mean antibody titre was found in donors under the age of 40 years old. These findings are similar to those of an Indian study, which discovered a higher mean antibody titre in the younger age group (7). We also discovered a low mean antibody titre in 50-year-old male donors, which is consistent with the findings of de Franca et al. Furthermore, a few studies among Indian donors revealed consistent findings of decreased titre with increasing age (7, 18). From our findings, donors aged over 50 years old are safer than other age groups. A recent study in Japan demonstrated that age was a suppressor of antibody responses. They revealed that the antibody titre after the mRNA severe acute respiratory syndrome-coronavirus (SARS-CoV)-2 vaccine significantly reduces with ageing (26). During ageing, T-cells favour short-lived effectors cells but not memory T-cells or T-follicular helper responses, thus affecting B cells for the generation of antibodies (27).

Apart from gender and age, the ABO antibody titres are influenced by ethnicity, diet, lifestyle and environmental factors. The high titre of ABO antibodies in Asian and African populations has been suggested to be caused by mosquito bites and parasitic intestinal infections (28). Asian countries are known to have a high percentage of forest areas, with increased susceptibility to mosquito bites. The high titres of ABO antibodies can also be due to vaccination or following antigen exposure, such as pregnancy or blood transfusion (29). A rise in antibodies following vaccination is more in anti-A because of the A-like substance in hog stomach pepsin used in toxoid production (30).

Changes in lifestyle have also been linked to variations in antibody titre levels. An apheresis donor with a 3-year history of probiotic consumption was found to have high anti-B titres and cause HTRs in a Group B recipient. Probiotic formulations contain bacteria with B-like antigens that increased anti-B production when ingested by individuals lacking the corresponding antigen (30).

Antibody titre levels are also influenced by diet. In their study, Kannan et al. (18) discovered a significant association between diet type and high antibody titre (*P* = 0.018). Donors with vegetarian diets have a higher chance of obtaining high titre antibodies (61.3%) than those on mixed diets (48.0%). The antibody titre was also reported to decrease over time. A 15-year study of the Japanese population revealed a decrease in anti-A and anti-B titres. This was due to environmental and personal hygiene improvements, which resulted in fewer parasitic infestations and enteric bacterial infections (31).

Our study showed that many of our blood donors had IgG in their ABO antibodies. Moreover, we discovered that the majority of them, like the Thai population, had high IgG titres. Thai blood donors had high anti-A and anti-B IgG titres of 93.0% and 95.3%, respectively (11). Our study also found that female donors were more likely to have high anti-B IgG titres. With this, one expects to observe a higher incidence of HTR if a blood product was given from a female donor. A high titre in the female group is due to the influence of sex hormones on immunity. At physiological concentrations, oestradiol can stimulate B-cell antibody production, suggesting one possible mechanism of higher antibody responses in females (25).

In our study, the prevalence of ABO haemolysin antibodies was 3.5%. This result is lower than that reported by Khampanon et al. (11), who found a prevalence of 69.0% in Thai Group O blood donors. The low result is possible because of the use of stored plasma that affects complement levels. In India, the prevalence of anti-A haemolysin was 16.6% and that of anti-B haemolysin was 10.7% (28). A high prevalence of haemolysin has been reported in Nigeria, at 52.8% (32).

We observed that anti-B haemolysins were more common in male donors, with anti-B titres ranging from 32 to 256. Female donors, on the other hand, had more anti-A haemolysins, with anti-A titres ranging from 64 to 128. Similarly, Khampanon et al. (11) discovered that high anti-A haemolysin titres were associated with female donors (*P* = 0.05). Our studies showed a high prevalence of ABO haemolysin in the young group of blood donors (< 40 years old). A study at the National Blood Transfusion Centre in Abidjan, Cote d’Ivoire, also discovered that ABO haemolysin was most prevalent among young blood donors aged between 25 years old and 29 years old (*P* = 0.001) (33).

The majority of our haemolysin-positive blood donors also had high IgM and IgG titres. A study on an Indian population found that 62.8% of haemolytic sera had high IgG titres. This was attributed to tetanus toxoid injections, as 81.0% of blood donors reported receiving them, and 38% donated blood products within 1 year of being immunised (28).

A recent study found a good correlation between high anti-A and anti-B IgM titres with haemolysis (34). The high titre of IgM and IgG antibodies can cause complement-mediated red cell lysis (35). The IgM antibodies can bind to C1q and initiate a complement cascade, leading to membrane attack, complex formation and consequent cell lysis. The IgG antibodies, particularly the IgG1 and IgG3 subclasses, can also fix complement efficiently, thus causing mild to fatal HTRs (34). A recent study by Bastos et al. found that the IgG1 and IgG3 types of ABO antibodies correlate well with haemolysis (*P* ≤ 0.001) (34).

## Conclusion

Our Group O blood donors had a high titre of ABO antibodies and a high titre of IgG antibodies, but a low prevalence of haemolysins. Males aged over 50 years old are the best O donors for preventing HTRs, particularly in situations that require mismatch transfusion. We also recommend that a transfusion unit regularly screen the ABO antibody titre for younger female Group O donors. This should be performed with a single plasma dilution with a cut-off point of 64. Hence, any high titre blood products should be labelled and transfused only to O group patients.

## Figures and Tables

**Table 1:** Age-related distribution of anti-A and anti-B titre in male (*n* = 185) and female donors (*n* = 126)

Age (years old)	Anti-A titre		*P*-value	Anti-B titre		*P*-value
	
	< 64 *n* (%)	≥ 64 *n* (%)		< 64 *n* (%)	≥ 64 *n* (%)	
Male donors *n* = 185						
18–29	19 (30.2)	54 (44.3)		26 (33.8)	47 (43.5)	
30–39	20 (31.7)	38 (31.1)	0.87^a^	23 (29.9)	35 (32.4)	0.142^a^
40–49	11 (17.5)	15 (12.3)		16 (20.8)	10 (9.3)	
≥ 50	13 (20.6)	15 (12.3)		12 (15.6)	16 (14.8)	

Total donor	63 (34.1)	122 (65.9)		77 (41.6)	108 (58.3)	

Female donors *n* = 126						
18–29	21 (58.3)	50 (55.6)		16 (57.1)	55 (56.1)	
30–39	9 (25.0)	25 (27.8)	0.682^a^	9 (32.1)	25 (25.5)	0.814^a^
40–49	6 (16.7)	11 (12.2)		3 (10.7)	14 (14.3)	
≥ 50	0 (0.0)	4 (4.4)		0(0.0)	4 (4.1)	

Total donors	36 (28.5)	90 (71.4)		28 (22.2)	98 (77.7)	

Note:

a Pearson’s chi-squared test was applied

**Table 2:** The association of anti-A and anti-B titre with gender and age (*n* = 311)

	Anti-A titre		*P*-value	Anti-B titre		*P*-value
	
	< 64 *n* (%)	≥ 64 *n* (%)		< 64 *n* (%)	≥ 64 *n* (%)	
Gender						
Male	63 (34.1)	122 (65.9)	0.308^a^	77 (41.6)	108 (58.3)	**< 0.001** a
Female	36 (28.5)	90 (7.5)		28 (22.2)	**98 (77.8)**	
Age (years old)						
18–29	40 (27.8)	104 (72.2)	0.333^a^	42 (29.2)	102 (70.8)	0.297 a
30–39	29 (31.5)	63 (68.5)		32 (34.8)	60 (65.2)	
40–49	17 (39.5)	26 (60.5)		19 (44.2)	24 (55.8)	
≥ 50	13 (40.6)	19 (59.4)		12 (37.5)	20 (62.5)	

Note:

a Pearson’s chi-squared test was applied

**Table 3:** The prevalence of high and low IgG titre of anti-A and anti-B antibodies in group O donors (*n* = 209)

Descriptive	Prevalence *n* (%)	Descriptive	Prevalence *n* (%)
Total donor with IgG titre ≥ 64	124 (59.3)	anti-A only IgG ≥ 64	33 (26.6)
	anti-B only IgG ≥ 64	25 (16.1)	
	anti-A and anti-B IgG ≥ 64	66 (53.2)	
Total donor with IgG titre < 64	85 (40.7)	anti-A only IgG < 64	13 (15.3)
	anti-B only IgG < 64	20 (23.5)	
	anti-A and anti-B IgG < 64	52 (6.2)	

**Table 4:** The association of anti-A and anti-B IgG titre with gender and age

	Anti-A IgG		*P*-value	Anti-B IgG		*P*-value
	
	< 64	≥ 64		< 64	≥ 64	
Total donors (%)	65 (39.6)	99 (60.4)		72 (44.2)	91 (55.8)	
Gender						
Male	41 (41.8)	57 (58.2)	0.482^a^	53 (54.1)	45 (45.9)	0.002^a^
Female	24 (36.4)	42 (63.6)		19 (29.2)	46 (70.8)	
Age (years old)						
18–29	28 (38.8)	44 (61.2)	0.252^a^	31 (41.9)	43 (58.1)	0.082^a^
30–39	15 (30.6)	34 (69.4)		14 (32.6)	29 (67.4)	
40–49	13 (52.0)	12 (48.0)		15 (55.6)	12 (44.4)	
≥ 50	9 (50.0)	9 (50.0)		12 (63.2)	7 (36.8)	

Note:

a Pearson’s chi-squared test was applied

**Table 5:** Demographic detail on 11 donors with haemolysins

Donors ID	Age (years old)	Gender	Ethnicity	Haemolysin	Ig classes	ABO titres
1	26	Male	Malay	B	IgG	32
2	26	Male	Malay	A and B	IgG, IgM	32, 256
3	27	Male	Malay	B	IgG	128
4	28	Male	Malay	A	IgG	64
5	30	Male	Malay	B	IgM	128
6	40	Male	Malay	A	IgM	64
7	51	Male	Malay	B	IgG	32
8	60	Male	Malay	B	IgG	32
9	20	Female	Malay	A and B	IgG, IgG	64, 64
10	25	Female	Chinese	A	IgG	128
11	33	Female	Chinese	A	IgG	128
